# Evaluation of the treatment of halitosis with photodynamic therapy in older patients with complete denture: Protocol for a randomized, controlled trial

**DOI:** 10.1097/MD.0000000000016275

**Published:** 2019-07-05

**Authors:** Katia Llanos do Vale, Anna Carolina Ratto Tempestini Horliana, Sergio dos Santos Romero, Alessandro de Melo Deana, Marcela Leticia Leal Gonçalves, Raquel Agnelli Mesquita Ferrari, Sandra Kalil Bussadori, Kristianne Porta Santos Fernandes

**Affiliations:** a Postgraduate program in Biophotonics Applied to Health Sciences, Universidade Nove de Julho; b Postgraduate Program in Rehabilitation Sciences, Universidade Nove de Julho, UNINOVE, São Paulo, Brazil.

**Keywords:** complete dentures, halitosis, photodynamic therapy

## Abstract

**Background:**

Halitosis is the term used to define an unpleasant odor emanating from the mouth. However, no studies have evaluated the causes and treatment of halitosis in the population of older adults with denture.

**Methods:**

A randomized, controlled trial is proposed. The patients will be divided into 2 groups: G1: older adults who wear complete dentures and will be treated with tongue scraper (n = 20); G2 older adults who wear complete dentures and will be treated with PDT (n = 20). If the halitosis persists, the participants will be submitted to hygiene procedures for the mucosa and dentures. The evaluation of halitosis will be made before and after treatments, with OralChroma^TM^. If the halitosis is solved, the participants will return after 1 week for an additional evaluation. Oral Health Impact Profile (OHIP-14) will be administered by a calibrated examiner on the day the patient history is taken (baseline) and 1 week after treatment for halitosis.

**Discussion:**

This protocol will determine the effectiveness of photodynamic therapy regarding the reduction of halitosis in older adults with complete denture.

**Trial registration:**

This protocol was registered in ClinicalTrial.gov, under number NCT03960983. It was first posted and last updated in May 23, 2019. https://clinicaltrials.gov/ct2/show/NCT03960983.

## 1 Introduction

Halitosis is a term used to define a transitory or prolonged unpleasant odor emanating from the mouth or breath.^[1,2]^ The prevalence of this condition is not well established, but there are reports of 15% to more than 50% of the population worldwide,^[3–5]^ with a nearly threefold higher incidence among men compared to women, independently of age.^[6,7]^ There is a correlation between older age and bad odor with aging, resulting in increased odor intensity.^[8]^

Few studies have analyzed the prevalence of halitosis in the population of older adults, which is defined by the World Health Organization as individuals aged 60 years or older.^[9]^ The increase in older adults at medical and dental offices underscores the need for greater attention to the adverse health conditions that affect this age group, considering the relationship between halitosis and both oral health and systemic conditions.^[10]^ In Thailand, researchers report a high incidence of halitosis in older adults.^[11]^ In Turkey, this condition was identified mainly in older females who wear complete dentures.^[12]^

Halitosis can exert a negative impact on social aspects, thereby affecting one's quality of life.^[13]^ This condition has a multifactor etiology. It is estimated that the source is intraoral in 90% of cases, resulting from bacterial degradation, especially anaerobic Gram-negative bacteria (*Treponema denticula, Porphyromonas gingivalis, Tannerella forsythia*), which produce volatile sulfur compounds (VSCs) on different surfaces of the oral cavity, such as biofilm on the dorsum of the tongue and in periodontal pockets.^[6,14]^ The VSCs produced through the metabolism of Gram-negative bacteria are hydrogen sulfide (SH_2_), emanated mainly from the dorsum of the tongue, with values higher than 112 ppb indicative of halitosis, methanethiole (CH_3_SH), which is predominantly higher in periodontal pockets, with values up to 26 ppb considered normal, and dimethyl sulfide (CH_3_SCH_3_), which can be of either a periodontal origin or systemic origin (intestinal, hepatic or pulmonary) and has a very low perception threshold (8 ppb). VSCs not only contribute to halitosis, but also result from the formation of volatile aromatic compounds, such as organic acids, acetic acid, propionic acid and the amines cadaverine, and putrescine. The production of VSCs in the oral cavity also depends on local factors, such as saliva, a reduction in the concentration of oxygen in the oral cavity as well as bacterial proliferation and metabolism.^[5,13,15–17]^ Therefore, the conditions that favor the retention of bacterial biofilm (dentures) and poor hygiene constitute predisposing factors for halitosis.^[5]^ Total dentures may favor the appearance of halitosis.^[8]^ Total denture hygiene is related to age, sex, and nocturnal use.^[9]^ The number of denture wearers and individuals with poor oral hygiene is high in the population of older adults, which favors the occurrence of halitosis.^[18]^

Only 8% of cases of halitosis are caused by extra-oral factors. Respiratory, gastrointestinal, hepatic, endocrine, and metabolic disorders have been associated with this condition.^[5,6,13]^

Halitosis can be diagnosed with 3 methods: the organoleptic test, gas chromatography, and a portable gas analyzer.^[13,14,19]^ The gold standard for this diagnosis is gas chromatography.^[17]^ The Halimeter and Oral Chroma are electronic equipment capable of detecting some exhaled VSCs.^[17,19]^ Oral Chroma is a device that specifically identifies VSCs (hydrogen sulfide, methanethiol, and dimethyl sulfide) through a sulfur detector, analyzes breath, and volatile compounds produced by detritus (decomposing food scraps) on the tongue and in saliva, but does not detect other possible causes of halitosis.^[17]^

Treatment for halitosis is related to its etiology. When intraoral, conventional therapy involves a reduction in microorganisms with the use of mouthwashes with antiseptic characteristics combined with mechanical removal using tongue scrapers or brushes, as the dorsum of the tongue is one of the main sources of bacterial colonization.^[13,19–21]^

Antimicrobial photodynamic therapy (PDT) is a treatment option for reducing the amount of intraoral microorganisms in localized infections.^[22–26]^ With this method, there are no reports of bacterial resistance, there are no side effects, the oral microbiota is preserved and toxicity to humans is minimal.^[22]^ PDT consists of the combination of a photosensitizing agent and light at a wavelength of 630 to 830 nm in the presence of oxygen.^[27]^ Despite the advantages of this method, there are no well-designed studies on PDT for the treatment of halitosis in older patients.^[28]^ Only 2 studies have addressed the treatment of halitosis with PDT in healthy adolescents and patients with multiple sclerosis,^[25]^ achieving favorable results. No studies have investigated the effects of PDT for the treatment of halitosis in older adults with denture.

The aim of the proposed study is to evaluate the treatment of halitosis using PDT or a tongue scraper in older adults with complete denture, as this population has not previously been studied.

## 2 Methods/design

A single-center, randomized, controlled, single-blind clinical trial was designed in accordance with the criteria recommended for interventional trials in the SPIRIT Statement. The project for the proposed study received approval from the Human Research Ethics Committee of *Universidade Nove de Julho* (certificate number: 12416619.1.0000.5511). This protocol was registered in ClinicalTrial.gov, under number NCT03960983. It was first posted and last updated in May 23, 2019.

### 2.1 Selection of individuals – characterization of sample

Two groups will be composed of older adults (60 years or older) in treatment at the dental clinic of *Universidade Nove de Julho.* Since they are already in treatment in the clinic, this will make recruitment simpler. After verbal and written explanations of the study, those who agree to participate will sign a statement of informed consent approved by the Human Research Ethics Committee of *Universidade Nove de Julho*. The study will be conducted in compliance with the precepts stipulated in the Declaration of Helsinki (revised in Fortaleza, Brazil, 2013).

### 2.2 Calculation of sample size

To calculate the sample size we established an error *err* = *x*_1_−*x*_2_ where *x*_1_ and *x*_2_ are the mean values of groups G1 and G2, whose variances are 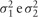, respectively. The effect size was calculated by: 
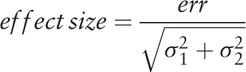


If the normal distribution hypothesis is rejected, the sample size should be corrected by approximately 5%.

**Figure 1:**
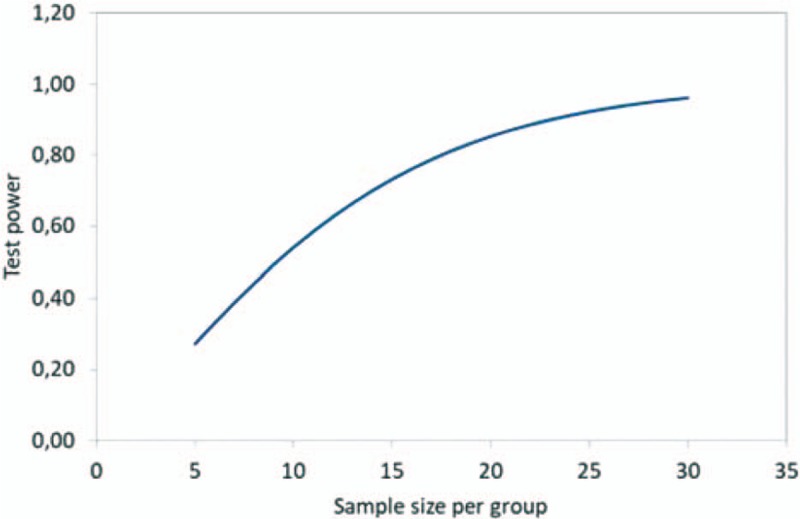
Sample size graph.

Observing statistical samples from the reference Mota et al^[14]^; to estimate the mean values and sample variance we obtain the following sample sizes for each group. G1: 18; G2: 18 (Fig. 1).

### 2.3 Inclusion criteria

Men and women aged 60 years or older using complete dentures.

### 2.4 Exclusion criteria

Dentate patients and edentulous with no complete denture hypersensitivity to the photosensitizing agent used in PDT, H_2_S < 112 ppb.

### 2.5 Training and calibration of examiner

An examiner (gold standard) will perform training and calibration exercises to maximize the reproducibility of the measurements. For such, 10 individuals with halitosis will be evaluated using the Oral Chroma device. These individuals will not participate in the main study. The intraclass correlation coefficient (ICC) will be calculated for the determination of intra-examiner agreement (≥0.90) with regard to the halitosis readings.

### 2.6 Randomization

**Figure 2:**
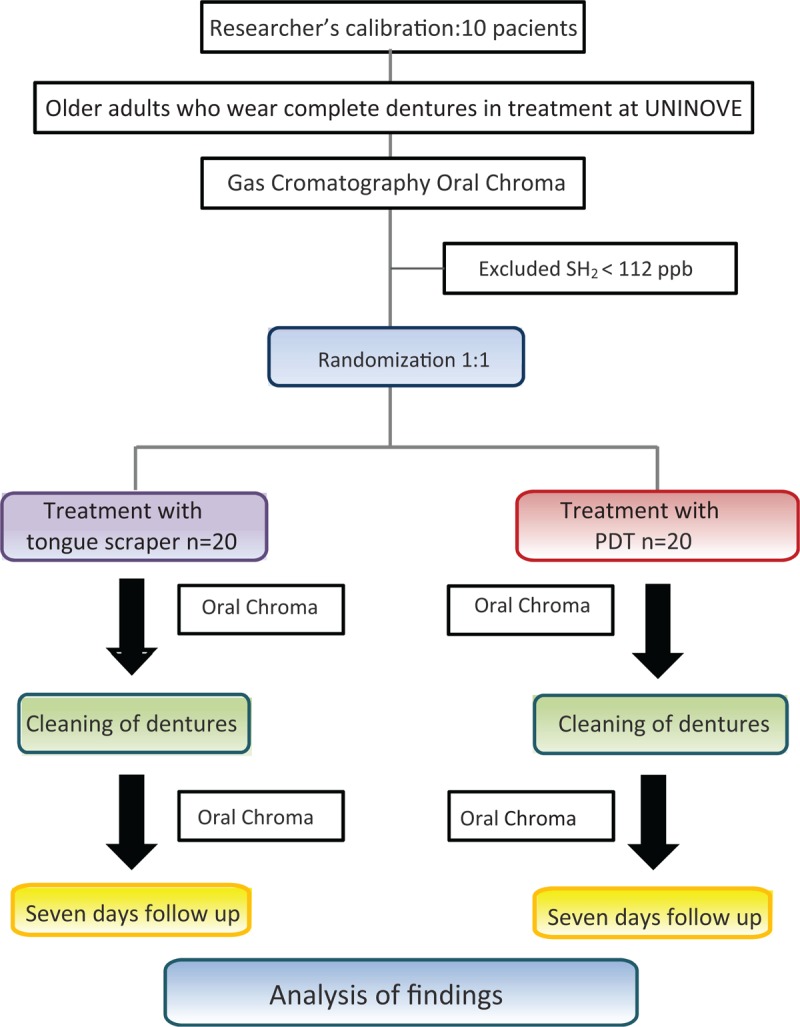
Activity flowchart.

The 40 individuals with complete denture will be randomized in 2 groups: Group A (20 individuals submitted to treatment with a tongue scraper) and Group B (20 individuals submitted to treatment with PDT) (Fig. 2). Opaque envelopes will be identified with sequential numbers (1–40) and will contain pieces of paper with the information of the corresponding experimental group (A or B). Blocked randomization will be performed in blocks of 5 patients (8 blocks for both treatments; example of a block: AABAB). The envelopes will be sealed and kept in numerical order in a safe place until the time of the treatments. The randomization and preparation of the envelopes will be performed by a researcher who will not otherwise participate in the study. Randomization will be performed using Microsoft Excel, version 2013.

### 2.7 Characterization of the study

**Figure 3:**
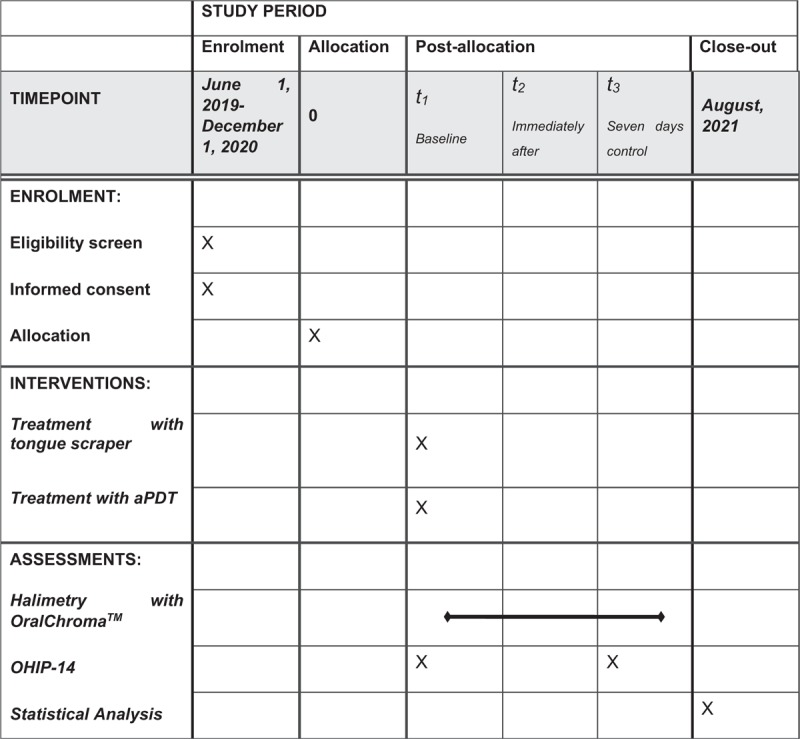
SPIRIT figure.

The experimental design will consist of 2 groups: G1-older adults with halitosis (SH2 ≥ 112 ppb) who wear complete dentures and treatment with a tongue scraper (n = 20); G2-older adults with halitosis (SH2 ≥ 112 ppb) who wear complete dentures and treatment with PDT (n = 20). The evaluation of halitosis will be performed at baseline (1st session), after treatment with a tongue scraper or PDT (1st session) and after 1 week (2nd session) (Fig. 3).

### 2.8 Patient history

After filling out a conventional questionnaire addressing the patient's general health, data will be collected on demographic (age, sex, marital status, occupation, schooling, living conditions, and income), medical (principal complaint, current state of disease, medical history, dental history, and use of medications), and behavioral (alcohol intake, eating habits, and oral hygiene habits) characteristics.

### 2.9 Assessment of halitosis

This is the primary outcome of the study. The portable Oral Chroma^TM^ (Abilit, Japan) will be used for the assessment of halitosis, which is a highly sensitive semiconductor gas sensor. The participant was instructed to rinse with cysteine (10 mM) for 1 minute, with the aim of selecting the gases produced by bacteria degradation. A syringe will be placed in the patient's mouth with the plunger completely inserted and the participant will breathe through the nose with the mouth closed for 1 minute, being careful not to touch the tip of the syringe with the tongue. The plunger will then be withdrawn to fill the chamber with air. This procedure will be repeated. The tip of the syringe will be cleaned to remove the saliva. The gas injection needle will be placed on the syringe and the plunger will be adjusted to 0.5 ml. This air will be injected into the input of the device in a single motion. When connected to a computer, the specific software accompanying Oral Chroma^TM^ produces a graph with peaks and values corresponding to the concentrations of VSCs (from 0 to 1000 ppb) with considerable precision after a period of 8 minutes. The results are stored in both the program and the device. As it is a device, Oral Chroma^TM^ is a blind assessor.

To standardize the readings, the exam will be performed in the morning and the participants will be instructed to avoid the ingestion of some strong spices, onion, garlic, and alcohol as well as the use of an antiseptic mouthwash 48 hours prior to the exam. The participants will also be instructed to abstain from coffee, mints, chewing gum, oral hygiene products, and personal hygiene products with perfume (after shave, deodorant, creams, or tonic) and brush only with water on the day of the test as well as not eat anything at least 2 hours prior to the test.^[23–25]^

*Analysis of Oral Health Impact Profile* (OHIP-14 questionnaire): The OHIP-14 is a shortened version of the original OHIP questionnaire used for the evaluation of the impact of oral health on quality of life. The items are distributed among subscales (functional limitation, pain, psychological discomfort, physical disability, psychological disability, social disability, and social handicap). The OHIP-14 will be administered by a calibrated examiner on the day the patient history is taken (baseline) and 1 week after treatment for halitosis.

### 2.10 Treatment with tongue scraper on coated tongue

The patients in the group G1 will receive treatment with tongue scraper. It will be positioned on the posterior third of the dorsum of the tongue in the region of the vallate papillae and pulled with light pressure to the apex of the tongue, following the manufacturer's instructions. This procedure will be performed only once.^[29]^ The participant will then be instructed to rinse with 50 ml of water for 2 seconds and spit.

### 2.11 Photodynamic therapy for coated tongue

**Figure 4:**
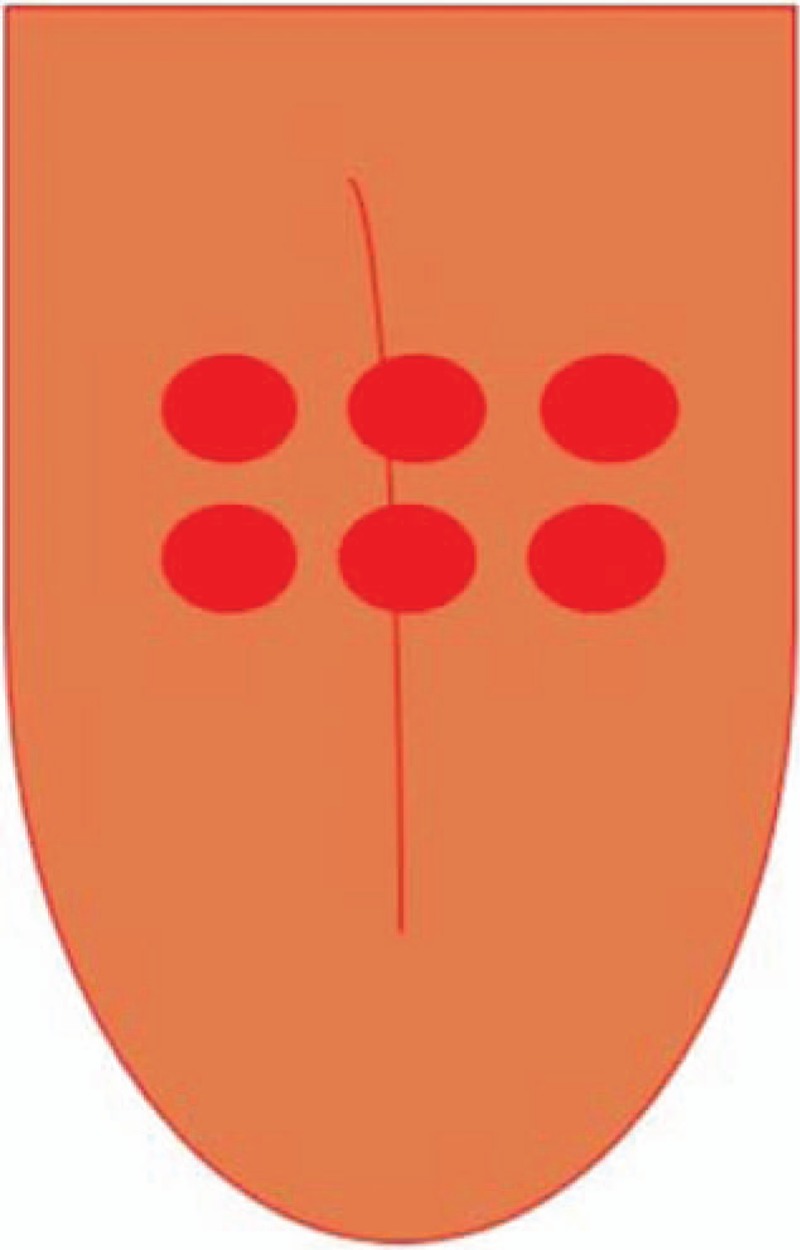
Irradiation spots in the tongue dorsum.

The patients in the group G2 will receive PDT. Methylene blue (0.005%) (Fórmula e Ação, São Paulo, Brazil) will be applied in sufficient quantity to cover the middle and posterior thirds of the dorsum of the tongue. After 5 minutes, irradiation will be performed with a red (λ = 660 nm) diode laser, operating with an output power of 100 mW, 9 J, radiant exposure of 320 J/cm^2^ and radiance of 3537 mW/cm^2^, using the point method in direct contact with the tongue (MM Optics Twin Laser, São Paulo, SP, Brazil).^[23–25]^ All parameters can be found in Table 1. Six points will be irradiated, with a distance of 1 cm between points (Fig. 4), considering the light spreading halo and effectiveness of PDT.^[24]^

**Table 1:**
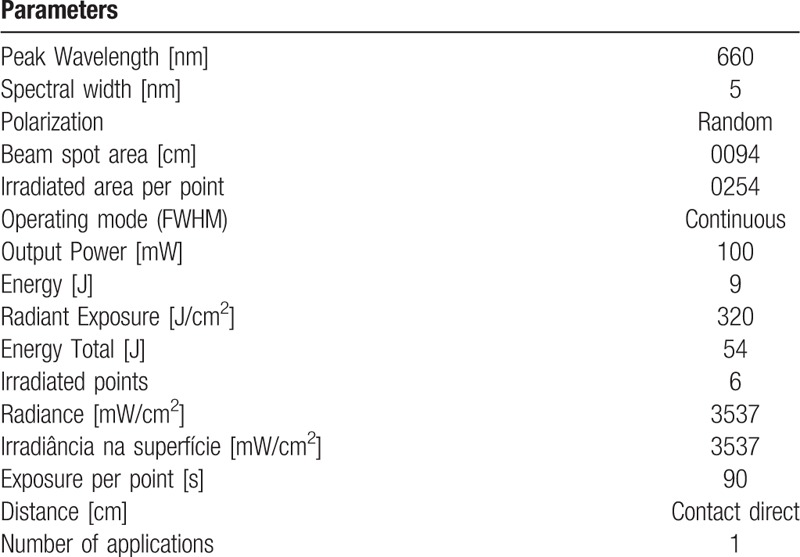
Dosimetric parameters used in each group.

During the application of the laser, both the patient and operator will use protective eyewear. The stained parts of the tongue will then be washed abundantly with saline solution until the complete removal of the dye.

### 2.12 Cleaning of dentures

The edentulous patients will be instructed to remove their dentures for cleaning. Sterile gauzes soaked in a 2% chlorhexidine solution will be applied over the entire inner and outer surfaces of the dentures. The mucosa of the edentulous patients will be cleaned with 0.12% chlorhexidine. After cleaning of the mucosa and dentures, patients will rinse with water for 1 minute.

### 2.13 Statistical analysis

The Lilliefors test will be used to determine the normality of the data. If normality is demonstrated, analysis of variance (*t* test student, Bioestat 5.3, Pará, Brazil) will be used to compare continuous and dependent variables among G1 and G.2. If non-normal distribution is demonstrated, Mann Whitney test will be used. The baseline data will be compared to the data collected at 1 week follow-up evaluation. A *P*-value < .05 will be considered indicative of statistical significance. The *χ*^2^ test will be used to compare categorical variables among G1 and G2, at baseline and the 1 week evaluation. These data will be expressed as mean ± standard deviation.

### 2.14 Strategies for obtaining the adequate recruitment of the participants

The participants will be recruited from the clinics of *Universidade Nove de Julho*. A researcher (KLV) will perform the exam to detect halitosis in a reserved room at the university. Treatment will be performed immediately after the diagnosis. The participants will be counseled with regard to oral health and will receive a set of instructions for performing daily oral hygiene. If they wish to withdraw their consent, there will be no loss for them. It is expected that patients will complete their participants, due to the benefit of halitosis reduction. Data from those who discontinue will not be used for analysis. After 1 week, all patients will be asked to return to *Universidade Nove de Julho* for the reevaluation of halitosis.

## 3 Discussion

Through the proposed clinical trial, we will evaluate the effect of PDT on halitosis in older adults who wear complete dentures, comparing this therapy to the use of a tongue scraper. PDT is an alternative therapeutic intervention for halitosis that diminishes the quantity of intraoral microorganisms, offers low toxicity,^[5]^ does not induce bacterial resistance, does not have side effects and preserves the oral microbiota.^[22]^

Halitosis is a common condition among older adults in nursing homes due to their greater frailty, poorer hygiene, greater risk of multi-morbidities, impaired cognition, and impaired functioning.^[30]^ However, few studies have analyzed the prevalence of halitosis in the older population, which, according to the World Health Organization is composed of individuals 60 years of age or older. In Thailand, the incidence of halitosis is high in the older population,^[11]^ whereas halitosis was mainly identified in older women who wore complete dentures in Turkey.^[12]^ A significant number of older adults wear dentures and this number has increased mainly in developed countries, at least with regard to a complete upper denture.^[31]^ Dentures themselves favor bacterial colonization, especially when associated with poor hygiene,^[18,32]^ which is a consequence of the limited dexterity and reduction in visual acuity in the older population, making it difficult to maintain adequate oral hygiene, which is indispensable to esthetic quality and the maintenance of oral health.

The surfaces of complete dentures are subject to colonization by oral microbiota, which can undergo changes when an individual becomes edentulous, with an increase in the presence of acidophilic bacteria in the oral cavity.^[33,34]^ Few studies have addressed the oral microbiota in patients who use complete dentures. It is reported, however, that changes in this microbiota stem from factors such as age, systemic condition, changes in salivary flow, differentiated dietary habits, the use of medications, poor hygiene, and changes in salivary pH,^[31]^ which can increase the amount and proportion of microorganisms that favor the occurrence of halitosis.

Changes in general health have also been identified as etiological agents of halitosis of an extra-oral origin,^[5,6,13]^ such as lung diseases as well as gastrointestinal, hepatic, endocrine, and metabolic disorders.^[5,6,13]^ Some treatments for halitosis are well established, such as the use of a tongue scraper and mouthwash. However, tongue scrapers are not habitually used by Brazilians due to the discomfort caused to the papillae and the lack of a technique of standardized use.^[29]^ The tongue scraper used in the proposed study has soft bristles to avoid discomfort to the tongue and injury to the adjacent periodontium during use. Moreover, its anatomic design favors handling. The method employed will be the same as that described by Mota et al.^[24]^

The dosimetric parameters for PDT were chosen based on previous studies that report favorable results with the administration of this type of therapy.^[23–25]^ According to Mota et al,^[24]^ immediate positive results were achieved with PDT for the treatment of the dorsum of the tongue in adolescents, with a reduction in halitosis due to the reduction in the concentration of VSCs. However, as mentioned above, the effects of PDT on halitosis in older adults with complete denture have not yet been studied.

## Author contributions

KLV and SSR made substantial contributions to the conception and design as well as the acquisition, analysis, and interpretation of the data. AMD, SKB, MLLG, RAMF and KPFS were involved in drafting the manuscript and revising it critically for important intellectual content. ACRTH and KPFS gave final approval of the version to be published.

**Conceptualization:** Katia Llanos do Vale, Sergio dos Santos Romero.

**Data curation:** Katia Llanos do Vale, Sergio dos Santos Romero.

**Formal analysis:** Katia Llanos do Vale, Sergio dos Santos Romero.

**Investigation:** Katia Llanos do Vale, Sergio dos Santos Romero.

**Methodology:** Katia Llanos do Vale.

**Supervision:** Kristianne Porta Santos Fernandes.

**Writing – original draft:** Anna Carolina Ratto Tempestini Horliana, Alessandro Melo Deana, Marcela Leticia Leal Gonçalves, Raquel Agnelli Mesquita-Ferrari, Sandra Kalil Bussadori, Kristianne Porta Santos Fernandes.

**Writing – review & editing:** Anna Carolina Ratto Tempestini Horliana, Alessandro Melo Deana, Marcela Leticia Leal Gonçalves, Raquel Agnelli Mesquita-Ferrari, Sandra Kalil Bussadori, Kristianne Porta Santos Fernandes.

## References

[1] Scully C Halitosis. BMJ Clin Evid 2014;9:1305. PMC4168334 25234037

[2] Kapoor U Sharma G Juneja M Halitosis: Current concepts on etiology, diagnosis and management. Eur J Dent 2016;10:292. 27095913 10.4103/1305-7456.178294 PMC4813452

[3] Armstrong BL Sensat ML Stoltenberg Jill L Halitosis: a review of current literatureno title. J Dent Hyg 2010;84:65–74. 20359417

[4] Çoban Z Sönmez I Halitosis: a review of current literature. Meandros Med Dent J 2017;18:164–70.

[5] Bicak DA A current approach to halitosis and oral malodor- a mini review. Open Dent J 2018;12:322–30. 29760825 10.2174/1874210601812010322 PMC5944123

[6] Scully C Greenman J Halitology (breath odour: aetiopathogenesis and management). Oral Dis 2012;18:333–45. 22277019 10.1111/j.1601-0825.2011.01890.x

[7] Silva MF Leite FRM Ferreira LB Estimated prevalence of halitosis: a systematic review and meta-regression analysis. Clin Oral Investig 2017. 10.1007/s00784-017-2164-5 28676903

[8] Miyazaki H Sakao S Katoh Y Correlation between volatile sulphur compounds and certain oral health measurements in the general population. J Periodontol 1995;66:679–84. 7473010 10.1902/jop.1995.66.8.679

[9] World Health Organization. World report on ageing and health. World Health Organization; 2015.

[10] McDowell JD Kassebaum DK Treatment of oral and nonoral sources of halitosis in elderly patients. Drugs Aging 1995;6:397–408. 7647427 10.2165/00002512-199506050-00006

[11] Samnieng P Ueno M Shinada K Association of hyposalivation with oral function, nutrition and oral health in community-dwelling elderly Thai. Commun Dental Health 2012;29:117–23. 22482262

[12] Bekirluog N A Iftã IA Bayraktar K Oral complaints of denture-wearing elderly people living in two nursing homes in Istanbul, Turkey. Oral Health Dental Manag 2012;11:107–15. 22976570

[13] Aylikci B Çolak H Halitosis: from diagnosis to management. J Nat Sci Biol Med 2013;4:14. 23633830 10.4103/0976-9668.107255 PMC3633265

[14] Mokeem SA Halitosis: a review of the etiologic factors and association with systemic conditions and its management. J Contemp Dent Pract 2014;15:806–11. 25825113 10.5005/jp-journals-10024-1622

[15] Kazor CE Mitchell PM Lee AM Diversity of bacterial populations on the tongue dorsa of patients with halitosis and healthy patients diversity of bacterial populations on the tongue dorsa of patients with halitosis and healthy patients. J Clin Microbiol 2003;41:558–63. 12574246 10.1128/JCM.41.2.558-563.2003 PMC149706

[16] Tolentino EdeS Chinellato LEM Tarzia O Saliva and tongue coating pH before and after use of mouthwashes and relationship with parameters of halitosis. J Appl Oral Sci 2011;19:90–4. 21552707 10.1590/S1678-77572011000200002 PMC4243744

[17] Salako NO Philip L Comparison of the use of the halimeter and the oral chroma^TM^ in the assessment of the ability of common cultivable oral anaerobic bacteria to produce malodorous volatile sulfur compounds from cysteine and methionine. Med Princip Pract 2010;20:75–9. 10.1159/000319760 21160219

[18] Verran J Malodour in denture wearers: an ill-defined problem. Oral Dis 2005;11 Suppl. 1:24–8. 15752093 10.1111/j.1601-0825.2005.01083.x

[19] Bollen CML Beikler T Halitosis: the multidisciplinary approach. Int J Oral Sci 2012;4:55–63. 22722640 10.1038/ijos.2012.39 PMC3412664

[20] Cortelli JR Dourado M Barbosa S Halitosis: a review of associated factors and therapeutic approach. Oral Health Braz Oral Res Braz Oral Res 2008;22:44–54. 19838550 10.1590/s1806-83242008000500007

[21] Beekmans DG Slot DE Van der Weijden GA User perception on various designs of tongue scrapers: an observational survey. Int J Dental Hyg 2017;15:e1–8. 10.1111/idh.12204 26865433

[22] Gursoy H Ozcakir-Tomruk C Tanalp J Photodynamic therapy in dentistry: a literature review. Clin Oral Investig 2013;17:1113–25. 10.1007/s00784-012-0845-7 23015026

[23] Lopes RG de Godoy CHL Deana AM Photodynamic therapy as a novel treatment for halitosis in adolescents: study protocol for a randomized controlled trial. Trials 2014;15:1–7. 25394474 10.1186/1745-6215-15-443 PMC4236439

[24] Costa da Mota AC Franï¿½a CM Prates R Effect of photodynamic therapy for the treatment of halitosis in adolescents - a controlled, microbiological, clinical trial. J Biophotonics 2016;9:1337–43. 27248838 10.1002/jbio.201600067

[25] Gonçalves M Kalil Bussadori S Dadalti Fragoso Y Effect of photodynamic therapy in the reduction of halitosis in patients with multiple sclerosis: clinical trial. J Breath Res 2017;11:0–28. Available from: S%0Astacks.iop.org/1752-7163/11/i=4/a=046006?key=crossref.fa839c1ca7bb967818fa3584040fca82. 10.1088/1752-7163/aa8209 28742057

[26] Carrera ET Dias HB Corbi SCT The application of antimicrobial photodynamic therapy (aPDT) in dentistry: a critical review. Laser Phys 2016;26 (12.):10.1088/1054-660X/26/12/123001 PMC5687295 29151775

[27] Braun A Dehn C Krause F Short-term clinical effects of adjunctive antimicrobial photodynamic therapy in periodontal treatment: a randomized clinical trial. J Clin Periodontol 2008;35:877–84. 18713259 10.1111/j.1600-051X.2008.01303.x

[28] Kellesarian SV Malignaggi VR Al-Kheraif AA Effect of antimicrobial photodynamic therapy and laser alone as adjunct to mechanical debridement in the management of halitosis: a systematic review. Quintessence Int 2017;48:575–83. 28512650 10.3290/j.qi.a38264

[29] Pedrazzi V Sato S de Mattos M Tongue-cleaning methods: a comparative clinical trial employing a toothbrush and a tongue scraper. J Periodontol 2004;75:1009–12. 15341360 10.1902/jop.2004.75.7.1009

[30] Zellmer M Gahnberg L Ramberg P Prevalence of halitosis in elderly living in nursing homes. Int J Dent Hyg 2016;14:295–300. 27476817 10.1111/idh.12236

[31] Kulak-Ozkan Y Kazazoglu E Arikan A Oral hygiene habits, denture cleanliness, presence of yeasts and stomatitis in elderly people. J Oral Rehabil 2002;29:300–4. 11896849 10.1046/j.1365-2842.2002.00816.x

[32] Nalcaci R Baran I Oral malodor and removable complete dentures in the elderly. Oral Surg Oral Med Oral Pathol Oral Radiol Endod 2008;105:5–9. 18417390 10.1016/j.tripleo.2008.02.016

[33] Mantzourani M Gilbert SC Fenlon M Non-oral bifidobacteria and the aciduric microbiota of the denture plaque biofilm. Mol Oral Microbiol 2010;25:190–9. 20536746 10.1111/j.2041-1014.2009.00565.x

[34] Teles FR Teles RP Sachdeo A Comparison of microbial changes in early re-developing biofilms on natural teeth and dentures. J Periodontol 2012;9:1139–48. 10.1902/jop.2012.110506 PMC4041159 22443543

